# Multiple Threats to Child Health from Fossil Fuel Combustion: Impacts of Air Pollution and Climate Change

**DOI:** 10.1289/EHP299

**Published:** 2016-06-21

**Authors:** Frederica P. Perera

**Affiliations:** Columbia Center for Children’s Environmental Health, Environmental Health Sciences, Columbia University Mailman School of Public Health, New York, New York

## Abstract

**Background:**

Approaches to estimating and addressing the risk to children from fossil fuel combustion have been fragmented, tending to focus either on the toxic air emissions or on climate change. Yet developing children, and especially poor children, now bear a disproportionate burden of disease from both environmental pollution and climate change due to fossil fuel combustion.

**Objective:**

This commentary summarizes the robust scientific evidence regarding the multiple current and projected health impacts of fossil fuel combustion on the young to make the case for a holistic, child-centered energy and climate policy that addresses the full array of physical and psychosocial stressors resulting from fossil fuel pollution.

**Discussion:**

The data summarized here show that by sharply reducing our dependence on fossil fuels we would achieve highly significant health and economic benefits for our children and their future. These benefits would occur immediately and also play out over the life course and potentially across generations.

**Conclusion:**

Going beyond the powerful scientific and economic arguments for urgent action to reduce the burning of fossil fuels is the strong moral imperative to protect our most vulnerable populations.

**Citation:**

Perera FP. 2017. Multiple threats to child health from fossil fuel combustion: impacts of air pollution and climate change. Environ Health Perspect 125:141–148; http://dx.doi.org/10.1289/EHP299

## Introduction

Like the many-headed Hydra in Greek mythology, fossil fuel combustion inflicts a multitude of serious health and developmental harms in children through its emissions of toxic particles and gases and carbon dioxide (CO_2_), a co-pollutant that is a major driver of climate change. Each of the myriad pollutants released from the burning of fossil fuels is capable of exerting multiple and cumulative adverse effects, either directly or indirectly. The developing fetus and young child, and especially the poor, are most vulnerable to the impacts of both toxic air pollutants and climate change. Were we, like Herakles, to succeed in slaying the Hydra, we would reap significant benefits for children, including fewer cases of preterm births, low birth weight, cognitive and behavioral disorders, and asthma and other respiratory illness—all of which have been linked to toxic air pollutants—as well as less heat-related disease, malnutrition, infectious disease, physical trauma, mental ill health, and respiratory illness related to climate change. The benefits would occur immediately and play out over the long term, because exposure-related damage, disease, or impairment in early life can affect health over the life course and even potentially across generations.

**Figure 1:**
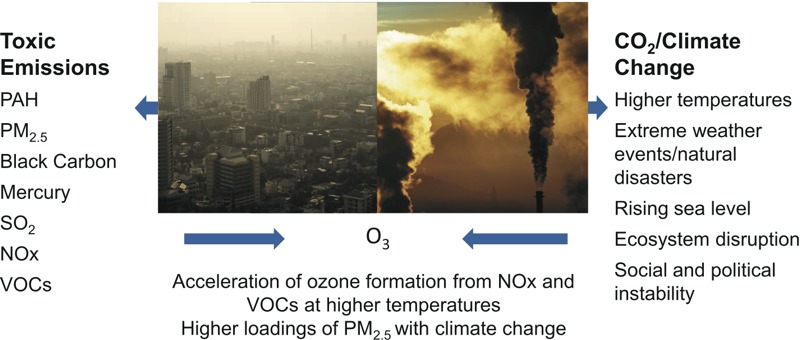
The burning of fossil fuels affects children’s health and development via toxic pollutants and climate change. NO_x_, nitrogen oxides. Photo sources from left: photo 1: Shutterstock (http://www.shutterstock.com/index-in.mhtml, http://www.shutterstock.com/license); photo 2: iStock (http://www.istockphoto.com/, http://www.istockphoto.com/legal/license-agreement).

**Figure 2:**
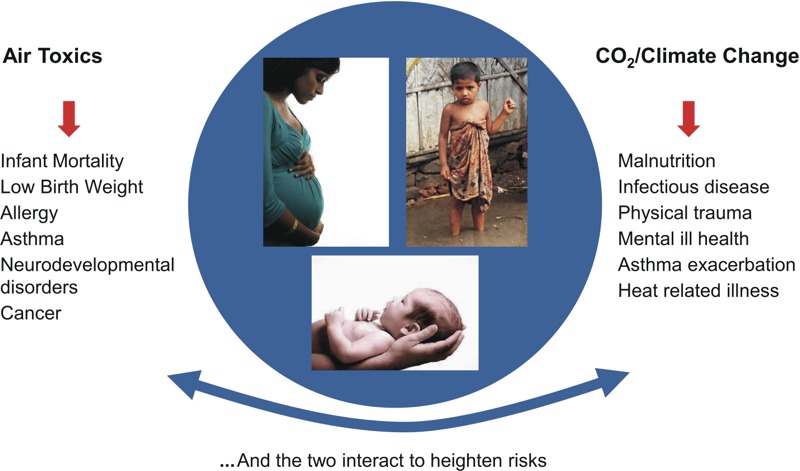
Multiple health impacts from the burning of fossil fuels. Photo sources, clockwise: photo 1: Thinkstock (http://www.thinkstockphotos.co.uk/, http://www.thinkstockphotos.co.uk/legal/license-information#); photo 2: Shutterstock (http://www.shutterstock.com/license, http://www.shutterstock.com/index-in.mhtml); photo 3: iStock (http://www.istockphoto.com/, http://www.istockphoto.com/legal/license-agreement).

The present commentary builds on earlier reviews (McMichael et al. 2006; Patz et al. 2005; Perera 2014; Schwartz 2004; Sheffield and Landrigan 2011; Xu et al. 2012) to summarize the current scientific evidence regarding the array of current and projected health and economic impacts in children from air pollution and climate change resulting from the burning of fossil fuels. The data alone provide a powerful argument for an integrated and child-centered air pollution and energy policy. However, going beyond the scientific and economic arguments, as Pope Francis has reminded us in his recent Encyclical “Laudato Si’” (Pope Francis 2015), is the powerful moral imperative to protect children and the less fortunate from toxic pollution and climate change. The pope’s message that global capitalism, based on the burning of fossil fuels, has created unsustainable consumption and shocking inequities within and between countries was also sounded in the recent report of the Lancet Commission (Watts et al. 2015). By highlighting the disproportionately heavy burden that falls on the very young, and especially the poor, who are most vulnerable to toxic air pollutants, as well as CO_2_-driven climate change from the combustion of coal, oil, and natural gas, the large body of evidence reviewed here calls for urgent and coordinated action to protect children. Figures 1 and 2 show the multiple combustion-related emissions and their associated impacts on the health of children, and hence the scope of the benefits that would result from decarbonization of the economy.

## Why the Focus on Early Windows of Development?

The developing fetus and young child are more biologically and psychologically vulnerable than adults to the many adverse effects of toxic air pollutants and physical trauma, psychosocial stress, nutritional deprivation, infectious agents, and heat waves associated with climate change from fossil fuel combustion. Contributing factors include children’s rapid growth, dynamic developmental programming vulnerable to dysregulation, immature detoxification, immune, and thermoregulatory systems, and their dependence on adult caretakers (reviewed by Bateson and Schwartz 2008; Sheffield and Landrigan 2011; Xu et al. 2012). The importance of protection during these early windows of development is indicated by the fact that most of the 86 billion neurons of the brain are formed during the prenatal period (Crelin 1973), and the brain, lungs, and immune system continue to develop during infancy through age 6 years and beyond (WHO 2006). In addition to their increased developmental vulnerability, children have greater exposure to toxic air pollutants than do adults (WHO 2006) and require three to four times the amount of food on a body weight basis than adults (Xu et al. 2012). Recent studies demonstrate that the fetal period and early childhood represent windows of susceptibility both to genetic damage (Perera et al. 2004) and epigenetic dysregulation from exposure to xenobiotics and stress (Dolinoy et al. 2007), with potential lifelong and transgenerational consequences (Heindel 2005).

The statistics attest to the differential vulnerability of the young and disadvantaged. According to the World Health Organization (WHO), one-third of the existing global burden of disease is caused by environmental factors: More than 40% of that burden is borne by children < 5 years of age, although they constitute only 10% of the global population (Smith et al. 1999; WHO 2002). Similarly, > 88% of the existing global burden of disease due to climate change also falls on children (Zhang et al. 2007), mainly in developing countries and populations of low socioeconomic status worldwide.

Air pollution and climate change disproportionally affect children in low income populations, both in the United States and globally (IPCC 2014a; Morello-Frosch et al. 2011). For example, low-income communities and communities of color in the United States have disproportionately high exposure to particulate air pollution and air pollution from coal-fired power plants (Bell and Ebisu 2012; Earthjustice 2011). In developing countries as well, there is a notable pattern of disproportionate exposure of the poor to air pollution (Mehta et al. 2014). Low-income populations are also most affected by climate change (Lynn et al. 2011; Stiglitz 2007). They often live in marginal areas most affected by floods or droughts. The effects of toxic exposures and climate change are magnified by inadequate nutrition, lack of adequate social support, and psychosocial stress due to poverty or racism (Wood 2003). The numbers of children living in poverty are staggering: 1.9 billion, of the total world population of 7 billion, are children < 15 years of age; and 1 billion of them are living in poverty (UNICEF 2015a). In the world’s most prosperous country, the United States, the child poverty rate is 22% (Annie E. Casey Foundation 2015). The striking socioeconomic inequalities that now exist in children’s health within and between countries will continue to grow under the projected trajectory of global fossil fuel consumption and climate change (Costello et al. 2009).

## Health and Developmental Impacts of Air Toxics

Fossil fuel combustion (coal, diesel fuel, gasoline, oil, and natural gas) for electricity production, heating, transportation, and industry is the main source of air pollution (U.S. EPA 2016). In 2011, globally, fossil fuels represented 82% of the total primary energy supply (World Energy Council 2013). In the United States, oil, natural gas, and coal account for 81% of current fuel use nationally (U.S. Energy Information Administration 2014). Frequently treated as a sidebar or “co-benefit” in articles on the mitigation of climate change, the burden of disease and impairment from air pollution is huge and increasing. The WHO reports that air pollution is the number one environmental health risk. In 2012, about 3.7 million deaths were attributable to ambient air pollution and 4.3 million deaths to household air pollution generated by indoor use of solid fuels (wood, charcoal, coal, crop wastes and dung) for cooking and heating (WHO 2016). The cumulative toll in terms of illness and impairment is likely to be even greater. Children represent the subgroup of the population most affected by air pollution and will be the primary beneficiaries of policies to reduce fossil fuel emissions over the next two decades (Cifuentes et al. 2001).

The emissions from the burning of fossil fuels include directly emitted fine particulate matter (PM), black carbon, polycyclic aromatic hydrocarbons (PAHs), mercury, nitrogen dioxide (NO_2_), sulfur dioxide (SO_2_), and carbon monoxide (CO), all of which have been associated with multiple health impacts. Sulfate and nitrates are secondarily formed from their respective gases and augment directly emitted fine PM. Ozone (O_3_) is created by photochemical reactions in the presence of sunlight involving its precursors—volatile organic chemicals (VOCs), CO, and NO_2_—reactions that are accelerated at higher temperatures. The best studied pollutants include deeply respirable particles having an aerodynamic diameter of ≤ 2.5 μm (PM_2.5_), PAHs, which are a class of hazardous air pollutants that includes known carcinogens and neurotoxicants, and O_3_, a strong respiratory irritant. Also emitted (but omitted here because of length limits) from burning or evaporation of fossil fuels are metals such as mercury and VOCs such as benzene. Most PM_2.5_, PAHs, and O_3_ and their precursors are emitted to the outdoor air; however, small particles and gases are able to penetrate from the outdoor into the indoor environment. Indoor sources include stoves and furnaces used for cooking and heating.

Early-life exposures to traffic-related pollution, PM_2.5_, PAHs, and O_3_ are associated with a multiplicity of effects on the developing fetus and child, which can have long-term consequences for child health.

### Adverse Birth Outcomes

Exposure to air pollutants is associated with low or reduced birth weight, small size for gestational age (SGA), and preterm birth (Choi et al. 2006; Ritz and Wilhelm 2008b). Black women exposed to particulate pollution had the greatest odds for all of the morbidity outcomes, most pronounced for very low birth weight (Salihu et al. 2012). Preterm birth and low birth weight are known risk factors for an array of neurodevelopmental disorders in children (Morse et al. 2009). Although the increases in risk from air pollution are generally modest (10–30% for preterm birth and low birth weight and between 5% and 20% for infant mortality) (Ritz and Wilhelm 2008a), the population exposed is very large (about 62 million women of reproductive age in the United States and 1.55 billion worldwide). In one of the few “natural experiments” tracking the health benefits of reducing air pollution, during the period when the Utah Valley Steel Mill closed down (August 1986–September 1987), particulate air pollution levels (measured as PM_10_; PM ≤ 10 μm) were reduced significantly (Pope 1996), and mothers who were pregnant around the time of the closure of the mill were less likely to deliver prematurely than mothers who were pregnant before or after (reviewed by Parker et al. 2008). The known or suspected mechanisms involved are reviewed elsewhere (Proietti et al. 2013).

### Respiratory Effects

In many studies, exposure to air pollutants, including PM_2.5_, O_3_, and NO_2_, in childhood has been clearly linked to reduced lung function. For example, a prospective study of almost 2,000 schoolchildren found that children exposed to higher levels of air pollution, including NO_2_ and PM_2.5_, had significantly lower lung function growth at 18 years of age, an age when the lungs are nearly mature and lung function deficits are unlikely to be reversed. Study children who had moved to new communities with lower particulate matter levels had increased growth in lung function, whereas subjects who had moved to communities with higher levels of particulate matter showed decreased lung function growth (Avol et al. 2001).

Exposures to ambient air pollution or traffic-related air pollution (O_3_, particulate matter, SO_2_, and/or NO_2_) are also well documented to exacerbate asthma in children (Tzivian 2011) and increase airway oxidative stress and airway inflammation in asthmatic children (Gasana et al. 2012; Kim et al. 2004; Li et al. 2012). Some studies have suggested a role of prenatal or early postnatal air pollution exposure in the development of asthma (Jung et al. 2012; Kim and American Academy of Pediatrics Committee on Environmental Health 2004). Prenatal or postnatal exposure to PAHs has been associated with decreased lung function in asthmatic children, asthma exacerbation, and possibly the onset of asthma itself (reviewed by Karimi et al. 2015). Long-term exposure to O_3_ is associated with deficits in lung function growth among schoolchildren (Rojas-Martinez et al. 2007), the prevalence of wheeze and allergic rhinitis, and the rate of newly developed sensitization to outdoor allergens (Kim et al. 2011).

Providing evidence of causality, during the Utah Valley Steel Mill closure, local improvements in respiratory disease (bronchitis and asthma), related hospital admissions, mortality, and school absenteeism paralleled the reduction in PM loadings (Pope 1989). Similarly, declining levels of NO_2_ and PM_2.5_, as a result of implementation of air quality–control policies in Southern California over a 13-year period, were associated with sustained improvements in lung function development of children over the same period (Gauderman et al. 2015). During the 2008 Beijing Olympics, when the central government temporarily restricted air pollution emissions in Beijing, statistically significant 27% and 25% reductions were observed in the mean concentrations of PM_2.5_ and black carbon, respectively (Rich et al. 2012; Wang et al. 2009), as well as a decrease in acute respiratory inflammation in children (Lin et al. 2011). The mechanisms by which air pollutants can affect children’s respiratory systems have been previously reviewed (Auten and Foster 2011; Huang et al. 2015; Klingbeil et al. 2014).

### Neurodevelopmental Effects

Data are more limited for neurodevelopmental effects than for birth outcomes and respiratory illness. However, air pollutants have been linked to an array of neurodevelopmental disorders in children. For example, in our cohort studies in New York City and Krakow, Poland, prenatal exposure to PAHs was associated with developmental delay, reduced IQ, symptoms of anxiety, depression, and inattention (Perera et al. 2012), ADHD (Perera et al. 2014a), and reductions in brain white matter surface in children (Peterson et al. 2015). We observed significant interactions between prenatal PAH exposure and material hardship on IQ (Vishnevetsky et al. 2015) and between prenatal PAH exposure and maternal demoralization on behavioral problems (Perera et al. 2013). Research in Tongliang, China, found that, compared with a cohort born before the closure of a centrally located coal power plant, a cohort conceived after plant closure had significantly lower cord blood levels of PAH–DNA adducts and higher levels of brain-derived neurotrophic factor (BDNF), a protein important in early brain development (Perera et al. 2008). A small study comparing school-age children in Mexico City with those in a less-polluted area of Mexico found that the cognitive deficits in highly exposed children matched the localization of the volumetric differences detected in the brain (Calderón-Garcidueñas et al. 2011). Other studies have linked roadway proximity or traffic particles to decreased cognitive function (Harris et al. 2015; Suglia et al. 2008). There is some emerging evidence that prenatal exposure to traffic-related air pollutants (Becerra et al. 2013; Volk et al. 2013) and PM_2.5_ (Raz et al. 2015) may be a risk factor for autism spectrum disorder (ASD). The suggested mechanisms involved in the neurotoxicity of air pollutants are reviewed elsewhere (Guxens and Sunyer 2012; Perera and Herbstman 2011).

In addition, respiratory illness associated with exposure to air pollution increases school absences, contributing to lower grades and test scores.

Finally, although the evidence on childhood cancer and air pollution is inconclusive, diesel exhaust particles and PAHs are known to be carcinogenic (ATSDR 1995). According to a recent review, exposure to residential traffic postnatally, but not prenatally, is associated with childhood leukemia (Boothe et al. 2014). Prenatal exposure to PAHs has been linked to chromosomal aberrations, a validated biomarker of elevated cancer risk in adults, in cord blood of New York City newborns (Bocskay et al. 2005).

## Health Impacts of Climate Change

The largest source of climate-altering greenhouse gas emissions (GHG) from human activities in the United States and globally is combustion of fossil fuels (coal, natural gas, gasoline, and diesel) for energy, electricity, heat, and transportation. The combustion by-product, CO_2_, is the most important GHG. In the United States, coal and natural gas are the biggest contributors to carbon pollution (one-third of all domestic carbon emissions). Methane is second in importance, released by production of natural gas, oil, and coal. Deforestation, agriculture, and landfills also produce CO_2_ and methane. Nitrous oxide and fluorinated gases contribute a much smaller fraction of GHG (U.S. EPA 2016). The present atmospheric concentrations of CO_2_, methane, and nitrous oxide are unprecedented in at least 800,000 years (IPCC 2014b).

The direct effects of climate change include increased illness, injury, and deaths from heat stress, floods, drought, and increased frequency of intense storms. The indirect effects are malnutrition and undernutrition, the spread of infectious disease vectors, food insecurity, illness due to increased air pollution and aeroallergens, and mental ill health from displacement and social and political instability (Perera 2014; Xu et al. 2012). In addition, population displacement from sea-level rise and conflict-associated displacement has numerous downstream impacts, such as increased rates of sexually transmitted infections due to social network disruption (McMichael et al. 2012). These direct and indirect impacts of climate change are compounded by those of air pollution.

Although there are few quantitative estimates of the proportion of childhood morbidity and mortality due to human-induced climate change, there is broad scientific agreement that both direct and indirect effects of climate change have already taken a significant toll on children, and the impacts are predicted to increase dramatically unless forceful action is taken. Children bearing the greatest burden of climate-sensitive diseases are those living in regions that have the lowest capacity to adapt to risks, but have contributed the least in global emissions of greenhouse gases (UNICEF 2015b).

### Malnutrition and Infectious Disease

Malnutrition and infectious disease represent the largest share of the burden of childhood morbidity and mortality attributed to climate change. The WHO estimated that climate change since the mid-1970s contributed to about 5 million lost disability-adjusted life years (DALYs) worldwide in 2000 through increases in diseases such as diarrhea, malnutrition, and malaria, mainly in children and in developing countries (Patz et al. 2005). The number of children affected is expected to rise to 175 million per year in the next several years (Save the Children UK 2007). In certain highland areas of Ethiopia and Colombia, malaria incidence has increased due to warmer temperatures (Siraj et al. 2014). Salmonella, a food-borne infectious disease, has become more prevalent across much of continental Europe as a result of higher temperatures (Patz et al. 2005). Their immature immune systems make children more susceptible to infectious disease pathogens (e.g., cholera and other diarrheal diseases) due to crop and water contamination from storms and floods. During early development, they are also more vulnerable to vector-borne diseases (e.g., malaria and dengue fever), which are likely to be increased in certain regions due to climate change (Patz et al. 2005). The Zika virus is the most recent addition to this list. Malnutrition places the young at further risk of such infectious diseases.

### Physical Trauma and Mental Health Impacts

Although no single extreme weather event, such as floods, droughts, bushfires, or hurricanes and cyclones, can be attributed entirely to climate change, there is agreement that the continuing patterns of global warming are contributing to the intensity and frequency of many such events (IPCC 2012; U.S. Global Change Research Program 2014). Weather-related disasters have directly affected an estimated 66.5 million children world-wide, 600,000 of whom died, every year from 1990 through 2000 (Pronczuk and Surdu 2008). Drowning is a major cause of fatality in children in developing countries. Sea-level rise due to global climate warming has made coastal storms increasingly dangerous for coastal infrastructure and inhabitants. According to a recent study, rates of sea-level rise between 1993 and 2011 exceeded by 60% the highest projections made in 2007 by the Intergovernmental Panel on Climate Change (IPCC) (Rahmstorf et al. 2012). Notable extreme weather events include the massive flooding across South East Asia in 2011 (UNICEF 2011) and Hurricane Katrina in 2005, which forced 1 million people in New Orleans from their homes and left 372,000 children without schools (Save the Children UK 2007; UNICEF 2011).

The psychological and emotional impacts of climate change include the acute or traumatic effects of extreme weather events and a changed environment; threats to emotional well-being based on direct experience or concern about future risks; chronic social and community impacts of heat, drought, migrations, and climate-related conflicts; and stress of post-disaster adjustment (Doherty and Clayton 2011; Patz et al. 2014). For example, high rates of anxiety and depression were found among children affected by Hurricane Katrina (Save the Children UK 2007). The mental health impacts are considered significant, but the effects in children as a vulnerable population have not been adequately described (Patz et al. 2014).

### Heat-Related Illness

A well-recognized direct effect of climate change is an increase in the frequency of deadly heat waves, such as that which resulted in 22,000–40,000 heat-related deaths in Europe in 2003 (Patz et al. 2005). Heat waves are predicted to become more frequent and severe in cities such as Chicago, Illinois, and Paris, France, with large increases predicted for western and southern United States and the Mediterranean region. New York City and Milwaukee, Wisconsin, may have three times their current average number of days hotter than 32°C (90°F) (Patz et al. 2014). Direct effects of heat waves on children include hyperthermia, heat stress, renal disease, and respiratory illness (Knowlton et al. 2009). During a deadly heat wave in California in 2006, children 0–4 years of age and the elderly were at greatest risk, with 2,500 children admitted to emergency rooms (Knowlton et al. 2009). Increased temperatures in areas of decreased precipitation are also resulting in the volatilization of persistent organic pollutants and pesticides, to which children’s developing nervous systems are particularly vulnerable (Sheffield and Landrigan 2011).

### More Illness from Increased Air Pollution and Allergens

An indirect effect of climate change is the increase in levels of certain toxic air pollutants. There is remarkable synergy between toxic air emissions from the burning of fossil fuels and climate change, in that higher temperatures due to climate change accelerate the formation of O_3_ from its precursors (VOCs, CO, and NO_2_). For example, O_3_ levels were substantially elevated during the European heatwave of summer 2003 (Schär et al. 2004). Climate change is predicted to elevate O_3_ levels over large areas in the United States and Europe, especially in the summer (Watts et al. 2015). Daily average O_3_ levels have been predicted to rise measurably across the eastern United States, with an estimated median 4.5% increase in summer O_3_-related deaths from climate change in the 2050s, compared with the levels of the 1990s (Knowlton et al. 2004). This seemingly small relative risk translates into a substantial attributable risk because many millions of adults and children are exposed. A median increase of 7.3% in summer O_3_-related emergency department visits for asthma in children 0–17 years of age is projected for the New York City metropolitan region by the 2020s (Sheffield et al. 2011). As reviewed above, in addition to increased mortality and asthma exacerbation, O_3_ is associated with decreased lung growth and lung function, as well as other respiratory problems in children.

Higher temperatures and greater CO_2_ concentrations also promote the growth of aeroallergens (e.g., pollens such as ragweed and mold), leading to more allergic disease and asthma in children (Shea et al. 2008). As noted earlier, aeroallergens can interact with O_3_ to increase sensitization and allergic asthma. Both ambient and indoor air quality are affected by these increases due to the easy penetration of pollutants into the indoor environment.

Concentrations of air pollutants such as PM_2.5_ are expected to increase because of changes in temperature, precipitation frequency, and air stagnation due to climate change (Watts et al. 2015). In addition, forest fires are increased by higher temperatures and lower soil moisture due to climate change, releasing large amounts of particulate matter, PAH, and black carbon. Given their small size, these pollutants can be transported hundreds of miles from their source, potentially exposing large numbers of children. As we have seen, these pollutants are associated with multiple adverse effects, most immediately respiratory symptoms, exacerbation of asthma, and chronic bronchitis in children (Xu et al. 2012).

Finally, both air pollution and climate change contribute to social and political instability. Children’s mental and physical health is adversely affected by forced migration and population displacement, perpetuating poverty and civil unrest in low-income and developing countries. These countries already bear most of the global burden of poverty and childhood disease; and children < 18 years represent 50% of their population (Sheffield and Landrigan 2011).

## Long-Term Health Impacts of Prenatal or Childhood Exposure to Air Toxics and Climate Change

Toxic air pollutants and climate change can affect health and functioning over the life course by launching a trajectory of adverse effects related to the initial physical or developmental impairment, and/or by “seeding” latent disease that becomes evident only in later life. For example, growth and developmental delays *in utero* associated with environmental exposures increase the risk for neurodevelopmental, respiratory, and other health problems in infancy and childhood, as well as heart disease, chronic obstructive pulmonary disease, and diabetes in adulthood (Ritz and Wilhelm 2008a). Prenatal exposure to certain environmental agents has also been linked to adult cancer (Heindel 2005). Developmental problems such as childhood ADHD (attention deficit/hyperactivity disorder) have been associated with early-life exposure to air pollution (Newman et al. 2013; Perera et al. 2014a). These problems may persist into adulthood, adversely affecting professional and personal life and increasing health care costs for individuals and families (Harpin 2005). There is considerable empirical evidence that early-childhood exposure to pollution affects not only health but also educational attainment and test scores (Currie et al. 2014). A reduction in child IQ can, in turn, have serious economic consequences in adulthood: Reduced IQ at 5 years of age associated with prenatal PAH exposure has been predicted to significantly lower lifetime earnings (Perera et al. 2014b). The impacts of climate change can also play out over a lifetime. For example, stunting of children’s bodies and brains due to malnutrition during the first 1,000 days results in lasting impaired functioning and reduced learning (Lake 2012). Early adversity and toxic stress are linked to later impairments in learning, behavior, and physical and mental well-being (Sandman et al. 2012; Shonkoff et al. 2012). This is illustrated by the finding that exposure early in pregnancy to elevated levels of cortisol, the so-called “stress protein,” resulted in significantly lower scores on measures of mental development in children (Sandman et al. 2012).

There is growing evidence of transgenerational impacts of early-life exposures to air pollutants, nutritional deprivation, and stress, possibly via the transmission of epigenetic changes (Champagne 2010; Heindel 2005). In humans, PAH air pollutants from the burning of fossil fuels have been shown to alter epigenetic marks in newborns, potentially affecting the regulation of genes involved in disease pathways, which may then be inherited transgenerationally (Perera and Herbstman 2011).

## Economic Benefits of Action

Lacking is a holistic assessment of the economic costs of the multiple health impacts of fossil fuel combustion on children’s health, hence the full economic benefits of action. First, estimates have largely been limited to a number of selected outcomes, mainly in adults, rather than on the multiple impacts on children and especially poor children as vulnerable populations. Second, a fundamental problem has been the lack of standardization in methods. For example, a range of economic outcomes has been used, such as cost per DALY averted, cost per quality-adjusted life year (QALY) gained, cost per case averted, cost per death averted, net cost or net present value, and economic benefits per unit of money invested (Hutton and Menne 2014). Further, widely varying outcomes, estimates of the value of life saved, and economic methods have been used, making it difficult to compare results among studies (Hutton and Menne 2014). For example, the value per life saved and the costs of health care in the United States are significantly higher than in most other countries and may not translate to lower-income countries. This has led to a patchwork of published estimates on air toxics or climate change. Despite these limitations, the available data indicate great economic benefits from coordinated reduction of pollution from fossil fuel combustion.

To give some examples for the United States: The estimated economic cost of preterm births attributable to airborne particulate matter in the United States in 2010 was $4.33 billion (Trasande et al. 2016). The cost of damages caused by air pollution in the United States was estimated to be at least $131 billion in the year 2011, with most of the cost attributable to health impacts (Jaramillo and Muller 2016). The U.S. EPA estimates the economic value of avoided premature deaths and health costs (benefits) attributed to the regulation of PM and other criteria air pollutants by the U.S. Clean Air Amendments will amount to almost $2 trillion for the year 2020—an amount that dwarfs the direct costs of putting those limits into effect ($65 billion) (U.S. EPA 2011).

The estimated monetary cost of the health impacts attributable to air pollution from existing coal plants in the United States in 2010 exceeded $100 billion per year (CATF 2010). Our study in New York City predicted that the increase in lifetime earnings for each cohort of newborns born to low-income mothers in New York City as a result of IQ gain from a hypothesized modest reduction of ambient PAH concentrations of 0.25 ng/m^3^ was $215 million per year (best estimate) (Perera et al. 2014b). Others have estimated the proportion of childhood illness from toxic environmental exposures more generally. In California, the environment contributes to an estimated 30% of the childhood asthma burden and 15% of the childhood cancer burden. Environmentally related costs of four childhood health conditions in California amount to $254 million every year and $10–13 billion over the lifetime of all children born every year (California Environmental Health Tracking Program, Public Health Institute 2015). Asthma had the greatest financial impact on an annual basis, costing families and the state > $208 million every year (California Environmental Health Tracking Program, Public Health Institute 2015).

The costs for non-U.S. countries are similarly large. The approximately 600,000 premature deaths and the diseases caused by air pollution in the WHO European Region in 2010 cost European economies an estimated US$1.6 trillion in 2010 (WHO Regional Office for Europe and OECD 2015). As recently reviewed in *Lancet*, the estimated cost of ambient air pollution in terms of the value of lives lost and ill health in OECD (Organisation for Economic Co-operation and Development) countries, plus India and China, is > US$3.5 trillion annually (Watts et al. 2015). The Clean Air for Europe (CAFE) Program estimates that achieving air quality targets for Europe has a benefit–cost ratio of between 6 and 19 (Hutton and Menne 2014).

The estimated health costs of climate change in the United States are also substantial. A recent study reported that six climate change–related events in the United States between 2000 and 2009 were associated with health costs of > $14 billion (95% due to premature mortality) and health care costs of $740 million (Knowlton et al. 2011). The estimated health benefits from air quality improvement, specifically lowered O_3_ and PM_2.5_ concentrations, under various CO_2_ mitigation policies in the United States are very large (from $140 to $290 billion), potentially offsetting the cost of U.S. and international carbon policies by as much as 10-fold (Thompson et al. 2014).

Globally, the WHO estimated that climate change contributed to > 150,000 deaths and 5.5 million lost DALYs in 2000 alone (McMichael and Butler 2004). According to WHO, the estimated cost of climate change from deaths and diseases such as diarrhea, malnutrition, malaria, and heat stress will be US$2–4 billion per year by 2030 (WHO 2015). The cost of acute and chronic mortality attributed to air pollution from climate change was estimated at €125 billion (US$141 billion) in 2050 for the 27 European Union (EU) countries (Hutton and Menne 2014).

Benefits of action are correspondingly large. In the EU, reduced air pollution from policies to mitigate climate change could deliver benefits valued at €38 billion (US$42 billion) each year by 2050 through reduced mortality alone; and moving to a low-carbon economy could reduce the control costs of non-CO_2_ air pollutants by ¤50 billion (US$56 billion) by 2050 (Watts et al. 2015). Up to US$230 billion of avoided external health costs annually by 2030 could be accomplished with an increase to 36% renewables in global energy consumption by 2030 (Watts et al. 2015). A study in Shanghai, China, estimated that, compared with the base case scenario, implementation of various low-carbon energy scenarios could bring health benefits worth US$2.6–6.2 billion (mid-value) in 2020 (Chen et al. 2007). In Taiyuan, China, policies to reduce coal burning emissions were correlated with a reduction in the ambient levels of air pollution and a 57% reduction in DALYs, with substantial estimated economic benefits (Tang et al. 2014). This summary underscores the need for comprehensive economic analyses of the full present and future benefits to children of reducing reliance on fossil fuels.

## Conclusion

A full accounting is needed of the mounting health and economic costs of pollution and climate change from fossil fuel combustion that are borne by children, so as to spur the concerted global mitigation efforts required. This accounting must address growing regional and socioeconomic disparities and the escalating threat to future generations. It is generally agreed that, unless strong action is taken now, our children and their progeny will inherit an unsustainable world, lacking the necessary ecological resources and services to support them (Yohe et al. 2007). The data reviewed here call for a harmonized clean air and climate change policy (Thurston 2013) that addresses the biological vulnerability of the young to the full array of physical and psychosocial stressors resulting from pollution from fossil fuels. The politically powerful value of protecting the health and well-being of present and future children is shared by all cultures and communities (Schene 1998; United Nations World Commission on Environment and Development 1987) and provides a strong lever for action.
